# Being in limbo: Women’s lived experiences of pregnancy at 41 weeks of gestation and beyond – A phenomenological study

**DOI:** 10.1186/s12884-017-1342-4

**Published:** 2017-06-02

**Authors:** Anna Wessberg, Ingela Lundgren, Helen Elden

**Affiliations:** 1 Institute of Health and Care Sciences, Sahlgrenska Academy, University of Gothenburg, Box 457, SE-405 30 Gothenburg, Sweden; 2 Sahlgrenska University Hospital, Diagnosvägen 15, SE-416 85 Gothenburg, Sweden

**Keywords:** Experiences, Lifeworld, Phenomenology, Post term pregnancy, Prenatal care

## Abstract

**Background:**

Globally, the prevalence of post term pregnancy (PTP) is about 5-10%, but the rate varies considerably between and within countries. PTP is defined as a pregnancy ≥294 days, but the definition is arbitrary. Many studies focusing on the prevalence, risks and management of PTP include pregnancies ≥41 gestational weeks (GW). However, qualitative interview studies concerning women’s experiences of PTP are lacking. Therefore, the aim of this study was to describe women’s lived experiences of a pregnancy ≥41 GW.

**Method:**

The study has a lifeworld research approach. Individual in-depth interviews were conducted from August 2013 to September 2014 with 10 healthy women with an expected normal pregnancy at GW 41 + 1-6 days in Gothenburg, Sweden. Interviews were conducted at the antenatal clinic or in the woman’s home, depending on her preference. Data were analysed with a phenomenological reflective lifeworld approach.

**Result:**

The essence of women’s experiences of a pregnancy at GW ≥ 41 was described as being in limbo, a void characterised by contradictions related to time, giving birth and the condition. Exceeding the estimated date of childbirth implied a period of up to 2 weeks that was not expected. The contradictory aspect was the notion that time passed both slowly and quickly. Negative feelings dominated and increased over time. The women experienced difficulty due to not being in complete control, while at the same time finding it a beneficial experience. Health care professionals focused solely on the due date, while the women felt neither seen nor acknowledged. Lack of information led to searches in social media. Previously, they had trusted the body’s ability to give birth, but this trust diminished after GW 41 + 0. In this state of limbo, the women became more easily influenced by people around them, while in turn influencing others.

**Conclusions:**

Being in limbo represents a contradictory state related to time and process of giving birth, when women need to be listened to by healthcare professionals. An understanding of the importance of different information sources, such as family and friends, is necessary. It is vital that women are seen and acknowledged by midwives at the antenatal clinics. In addition, they should be asked how they experience waiting for the birth in order to create a sense of trust and confidence in the process.

## Background

The World Health Organisation (WHO) has defined post term pregnancy (PTP) as lasting 294 days or longer, i.e., gestational week (GW) 42 + 0 days, from the first day of the last menstrual period [1, 2]. However, there is no consensus on this definition. Studies analysing the risks and management of PTP also include pregnancies from ≥41 GW, i.e., late term pregnancy. There is no known aetiology of PTP. Some identified causes are foetal anencephaly, foetal adrenal hypoplasia or insufficiency and placental sulphates deficiency. Risk factors include primiparity, advanced maternal age, maternal obesity, heredity, previous PTP and a male foetus [3–7].

The prevalence of PTP is approximately 5-10% [8] but varies between countries. Factors that influence prevalence are population characteristics such as maternal age, rate of preterm birth e.g. <37 GW, interventions, induction of labour, availability of routine ultrasound pregnancy dating, the number of primiparous women in the population and pregnancy surveillance routines [9]. PTP prevalence varies between 0.4% in Austria and 8.1% in Denmark [10–12]. The reported PTP prevalence in Sweden was 6.9% in 2013 [13]. Previous research has focused on the prevalence and risk of perinatal complications in PTP. Such complications increase the risk of foetal mortality, meconium aspiration syndrome (MAS), umbilical cord complications, asphyxia, pneumonia, sepsis, convulsions, shoulder dystocia, traumatic injuries, peripheral nerve damage and neonatal encephalopathy [5, 14, 15]. Furthermore, developmental delay [16], increased rates of obesity and early markers of metabolic syndrome have also been reported in post term children [16–18]. Maternal complications in PTP are puerperal infections, postpartum bleeding, disproportion, labour dystocia, emergency Caesarean section and cervical lacerations [5].

Due to the increased risk associated with PTP, a solution would be to suggest induction of labour before the pregnancy becomes post term. However, most randomised controlled trials (RCTs) comparing induction with awaiting the spontaneous onset of labour, with or without foetal surveillance, are small and of poor methodological quality. An HTA meta-analysis including 17 RCTs (*n* = 7223 women) [19], and recently published systematic reviews including 31 RCTs [20] and 157 RCTs [21] showed a lower perinatal mortality rate in the induction group, fewer cases of MAS and either no difference or a lower risk of Caesarean section after induction, compared to spontaneous labour. A methodological problem in individual studies and systematic reviews is that critical outcomes such as perinatal mortality and hypoxic ischemic encephalopathy are rare [22–24]. Thus, very large studies are needed to address such outcomes. Thus, there is no consensus as to the optimal time for labour induction or the optimal method of antenatal surveillance. However, according to guidelines in Norway (Norwegian Directorate of Health) [25], Denmark (The Danish Society of Obstetrics and Gynaecology, DSOG 2011) [26], UK (The Royal College of Obstetricians and Gynaecologists, RCOG) [27], USA (The American College of Obstetricians and Gynaecologists, ACOG) [28], and Canada (The Society of Obstetricians and Gynaecologists of Canada, SCOG) [29], induction should be initiated between GW 41 + 0 and 42 + 0.

It is well known that women’s experiences of childbirth constitute an individual, complex life event, related to the outcome for the child and the mother [30–32]. Studies reveal that women’s anxiety and worries increase when the pregnancy extends beyond GW 41 + 0 [33, 34]. Three cross-sectional studies [31, 35, 36], one RCT [37], one cohort study [31] and two qualitative studies [38, 39] all discuss experiences of induction of labour. Two cross-sectional studies from Sweden with 1111 and 936 women, respectively [31, 36], showed that induction of labour, irrespective of the cause, was associated with a more negative experience of childbirth. This finding is not supported by a study from Nigeria (*n* = 252 women) [35] and an RCT from Norway (*n* = 508 women) [37], which demonstrated that a majority of the women expressed satisfaction with the induction process. Only one RCT assessed women’s experiences of induction or expectant management policy (*n* = 508 women) [37]. At the time of randomisation, 74% would have preferred induction if given the choice. The majority of the women (84%) who were induced had a positive childbirth experience. Nevertheless, induction led to a more intensive labour. Studies have showed that women lack information and are thus unable to make an informed decision about the induction process [37–39].

To date there is only one interview study of women’s experiences of a PTP. This study found that the focus on medical issues and the categorisation of the pregnancy based on medical statistics lead to the women feeling less involved during their PTP. The women described a feeling of being pathological patients and expressed a desire to be induced earlier to avoid stigmatisation by relatives and friends [40]. Thus, there is a need for more research about women’s experiences of a PTP. The aim of this study was to describe women’s lived experiences of a pregnancy ≥41 GW.

## Methods

A qualitative study with a phenomenological reflective lifeworld approach, as described by Dahlberg et al. [41] was conducted in order to illuminate the essential meaning of healthy women’s lived experiences of an expected normal pregnancy at ≥41 GW. A qualitative method is applied when there is limited knowledge of the phenomenon under study and can be particularly valuable for describing a phenomenon from the woman’s point of view. Reflective lifeworld research is an empirical method for research based on the work of Husserl, Gadamer and Giorgi [41]. The phenomenological approach is grounded in lifeworld research and used to describe and understand individuals’ experiences of different phenomena [41]. Lifeworld research with a phenomenological approach requires openness to the phenomenon on the part of the researchers, who must bridle their own preunderstanding, which includes knowledge and personal experiences [41]. The phenomenological approach was deemed most suitable for gaining a deeper understanding of PTP. The strength of this approach is that it provides a possibility to delve more deeply into the nuances and variations of a specific phenomenon. Variations were reached by interviewing women with different background, age, and parity.

In Sweden midwifes are the primary care givers for a healthy woman with an expected normal pregnancy, during pregnancy, labour and post-natal care. If complications occur during pregnancy and labour, an obstetrician will assume the main responsibility for the care. Antenatal care is normally provided by a midwife at a primary health centre. When the woman reaches GW 42 + 0 an appointment is arranged at the local hospital for induction of labour, where a hospital midwife will take care of the woman. Hence, there is a gap in the care chain, which can affect the information provided to the woman and her state of mind.

The present study was performed at an antenatal care unit in Gothenburg, Sweden, and an antenatal unit in a suburb of Gothenburg from August 2013 to September 2014. The inclusion criteria were a woman with an expecting normal single pregnancy in GW 41 + 0 to GW 41 + 6. Midwives gave eligible women oral and written information about the study at a regular check-up/appointment at their antenatal care unit. If the woman was interested, the midwife sent the woman’s telephone number by e-mail to the first author (AW), who in turn contacted the woman by telephone approximately 2-5 days before the interview. AW then explained the study in more detail and if the woman was still interested in participating, a time and place for the interview were arranged. The women received both oral and written information from the researcher, were informed about the purpose of the study and about the voluntary nature of participation. They were assured that the data would be treated confidentially and that they were free to withdraw at any time. The women in turn gave their written consent before the interview. Fourteen women were interested and booked for an interview but four women went into labour before the interview. The interviews were conducted at the antenatal unit or in the home, in accordance with the preference of each woman. An in-depth interview technique [42] was employed in order to create a relaxed, trusting and open atmosphere aimed at enabling the women to express their lived experiences of pregnancy ≥41 GW. There was an option to provide further support, but this was not requested by any of the participants. The following open-ended question was used: Can you tell me about your experiences of 41 completed GW? Follow-up questions were posed to gain a deeper understanding such as “can you describe more”. All interviews were conducted by the first author (AW), lasted between 25 and 50 min and were audiotaped and transcribed verbatim.

### Data analysis

The data were analysed based on phenomenological reflective lifeworld approach, as described by Dahlberg et al. [41]. First, the interviews were recorded and transcribed verbatim. Next step involved reading the transcript several times in order to gain an initial sense and understanding of the data in their entirety. Then meaning units were identified, i.e. parts of the text corresponded with the aim of the study. The meaning units were then grouped into different clusters. Nvivo 10 software (http://www.qsrinternational.com/) was used for extracting meaning units and grouping clusters. The last step involves describing the essence by moving between the clusters and back to the transcribed interviews. Seeing and understanding the essence as a new whole involved moving between the parts and the text as a whole. When the essence emerged, the next step was to describe it in depth by its constituents with all their nuances and variations. The analysis was an ongoing process, which meant constant and continuous checks to detect any inconsistency between the whole and the collected data. All quotations were translated from Swedish to English by a native speaker. During the analysis there was continual discussion in the research group in order to identify the essential structure and its constituents [41].

## Results

Ten women participated in the study. They were all married/cohabitating and varied in age from 23 to 37 years (mean 30.5 years). Three women had primary and secondary school degrees and seven women had university degree. All women were satisfied with their workplace/profession. Eight women were expecting their first baby and two women their second. All participating women spoke Swedish.

The essence of the phenomenon of women’s experiences of a late term pregnancy was described as being in a state of limbo, a void characterised by contradiction in relation to time, giving birth and the condition. Contradictions in connection with waiting evoked a negative feeling, as well as a form of restlessness, implying difficulties with simply being and taking each day as it comes as well as a sense of irritation and frustration. However, an advantage of the delay was gaining another day before the birth, which was sometimes experienced as positive. In their state of limbo, the women were more easily influenced by people around them, while in turn influencing other. There was also a feeling of insecurity concerning the body’s ability to give birth, which could increase due to opinions and advice from others. There was a belief that the child would arrive when it was ready, but that it preferred to remain in the womb. This entailed a contradiction in that the women did not want the labour to begin too quickly due to consideration for the unborn child, while at the same time they longed for her/his arrival.

The temporal contradiction implied that time passed both slowly and quickly. The estimated time of birth had passed, implying days and weeks that should never have even existed. The body was ready but the child was not; the body and child were not synchronised. Being in a temporal contradiction mainly implied negative feelings, even if initially this period was considered normal and had no significant effect on the pregnant women. As the days passed, more negative thoughts arose, despite the fact that the feeling about the last days of pregnancy and birth was basically positive. They found that just being was difficult, which in turn made them frustrated about the delayed birth.

The contradiction in relation to giving birth involved not only the will to give birth, but also trust and a desire to wait until the unborn child was ready. Thoughts became more negative, although positive thoughts and feelings still remained, including a sense of trust in the body’s ability to begin birth. The women were surprised by their own contradictory thoughts. The desire for a natural childbirth with a spontaneous labour onset began to change and they shifted from a positive view of trusting the body to initiate childbirth to mistrusting their bodies. Thoughts of inducing the birth began to arise, which included anxiety about their inability to achieve a natural birth without being induced, even in any future pregnancies.

Contradiction in relation to the women’s condition meant that healthcare professionals focused solely on the expected due date, while at the same time the women felt neither seen nor acknowledged by them. The due date dictated the planning of the visits to the midwife and the time for an eventual induction of labour, leaving no place for acknowledgement of the women. The essence can be further described by five constituents: “negative feelings and thoughts”, “difficulty associated with waiting for labour”, “unmet expectations”, “seeking alternative sources of information” and “the influence of others”.

### Negative feelings and thoughts

Waiting was dominated by negative feelings that increased over time, thus few negative feelings or thoughts arose during the first week following the scheduled due date. Negative thoughts were; disappointment, doubt, hesitation, frustration, irritation, impatience, destructive thinking and the belief that the delivery would never begin. Negative thoughts and feelings arose even when there were initial expectations that the delivery could be a week later than the due date*.* A strategy was to try to keep up their spirits and convince themselves that “all is well”. At the same time, there were always negative thoughts at the back of their minds, which became worse when yet another week of waiting began.

*You try to keep up your spirits anyway and convince yourself that everything is okay, but the emphasis is on the downside after the calculated date, so I feel that mentally it’s mainly negative. ID 3, primigravida.*

Part of the waiting implied dealing with negative thoughts and feelings of doubt about whether the delivery would start, while at the same time the women tried to convince themselves that the delay was normal. When planned activities in preparation for the due date were completed, the women felt that the child should soon arrive. Negative thoughts and feelings arose when the birth did not begin as planned.

*A feeling that nothing has happened, it will never come, this will continue day in and day out and I will wake up like this every day in some sort of limbo. ID 3, primigravida.*

According to the women, it was important that they did not focus solely on the pregnancy, or why the birth was delayed. One way to remain calm and avoid negative thoughts was to spread activities over several days so that there was something to do each day. Planning included both useful and practical activities as well as meeting friends and acquaintances in order to feel that their lives were going on as usual.

*I try to jot down (activities) mainly so that I say that tomorrow, right, I have to do this, and it’s almost good that I won’t give birth tomorrow so that I can do this beforehand, constantly ticking things off that are super important. ID 3, primigravida.*

The women described restlessness as a negative dimension that increased over time. One way to deal with and prevent negative thoughts was keeping busy, although even here there was a contradiction in terms of how much planned activity was beneficial. They were concerned that too much activity would drain their energy if labour began that night, while at the same time it was important to plan the day to include some form of activity.

*I feel so divided, because one second I feel it’s good to try to be active so as not to feel restless and to make the time pass more quickly, etc. But on the other hand, I should try to rest in case I go into labour during the day, otherwise I might not be strong enough for the delivery; you become ambivalent and don’t know what to do. ID 8, primigravida.*

Impatience was described as a negative feeling and implied a feeling of ambivalence in relation to time; time passed both slowly and quickly. The importance of doing pleasant things that they could manage became more evident during the days of waiting.

*…before, I did everything at the same time so that things went as quickly as possible, but now I no longer do things simultaneously because I want the time to pass slowly… or as quickly as possible…for that reason I kept busy last week and we had about a thousand cleaning projects you know, fixing the blinds and such things, and they’re finished now, so now…I don’t know, the week has gone by very slowly, but at the same time, quickly. ID 10, primigravida.*

Negative thoughts that increased over time involved concern for the coming child. There was even worry that there was something wrong with the unborn child, which was delaying the onset of labour. The question that was constantly on the women’s minds was whether there was a risk for the unborn child if the delivery did not start spontaneously on the scheduled day. These thoughts were present and increased over time despite efforts to suppress them.

*Just this not knowing...is something wrong with the unborn child because it doesn’t want to come out or can something happen if it doesn’t come out?… I try not to think about that because it’s destructive…you really try to stop thinking about it, but the fact that nothing is happening is there constantly at the back of your mind. ID 2, multigravida.*

The women described negative thoughts such as mistrusting the body’s ability to begin labour or the possibility that there could be something wrong with their bodies. They also reflected on their age and if it could be the reason that the labour had not begun. These thoughts and reflections on the reason why the birth process did not begin increased over time. A kind of contradiction emerged, whereby they saw the advantage of waiting, while also doubting and mistrusting their body’s ability to go into labour.

*The closer, yes, the closer it gets, the more worried I become…yes, because I begin to wonder; is there something wrong with me or is it because I don’t have that hormone and why don’t I have it and…will I need to be induced in all pregnancies? ID 4, primigravida.*

While negative thoughts dominated, some described a feeling of trust that the child was well and that there was no need to worry about if, when and how the labour would begin.

*I haven’t actually thought so much about it because I feel it’s well…or at least according to all the check-ups and everything else I’ve been through... I have really thought that it isn’t that I’m afraid of inducing the birth because the baby would be unwell, or that [the baby] is unwell now, or that I am overdue. I haven’t thought about it at all, but I think [the baby] is well. ID 8, primigravida.*

### Difficulty associated with waiting for labour

Difficulty associated with just being was related to the need for control; control over work, meetings, planning and life in general. The need for control and be able to plan was associated with how society functioned. Lack of control over what was about to happen and when the birth would take place was experienced as difficult. Nevertheless, the women described a slight loss of control and listening to the signals from the body as healthy. During the period of waiting the need to be in control while at the same time being unable to influence the start of labour was put to the test. The women stated that unlike pregnancy, which could be planned, the time of birth could not, which they considered understandable, but still found difficult to accept.

*No, I have no control over this and I cannot start it… it creates a very difficult feeling inside ...of course it would be nice to have control and get it started. ID 4, primigravida.*

The women described that there are constant thoughts about and comparisons of how society functioned in the past and today. The feeling was that in today’s society everything has to be perfectly planned, while in the past things happened naturally and it was not difficult to just be, as people accepted that this was the way things were. The women reported that it was easy to find information about almost everything on the Internet and although they knew that the information was not always credible, they still found it difficult to refrain from searching for it. They said that both positive and negative information made it more difficult for them to trust their body. Communicating through different websites also made the women feel more stressed.

*Yes, I often think so; before, this was completely natural, it’s only now that people get completely stressed out by everything. That’s modern society, everything must be perfect. ID 8, primigravida.*

Just being and waiting for the labour to begin was challenging and their feelings and thoughts concerned the body’s ability to initiate labour, which made them look for signs that the labour was under way and try to maintain hope that it would be a normal birth. The waiting period was dominated by the desire for a spontaneous onset of labour and that the birth would be as normal as possible. There was a belief that labour usually begins at night.

During the whole pregnancy, focus was on the due date, which implied that in a way the time after this date did not exist. Despite this, there was also an awareness that few women actually gave birth on the calculated day.

*You are so set on a fixed date, despite knowing that very few deliver on…the due date, but it’s still somehow the only thing on one’s mind. ID 1, primigravida.*

The women described thinking of how difficult it could be after the child was born, which led to positive feelings and thoughts.

*One should be grateful for…all of these days... so it makes the negative seem positive. ID 3, primigravida.*

Waiting led to differing, sometimes unexpected moods, which implied that occasionally the women did not recognize their reactions and surprised themselves. They stated that they understood that it was normal to be overdue, but being in that situation led to a strong feeling of frustration and irritation that the labour had not started.

*You prepare yourself every day for something to happen soon and then it doesn’t, which is a bit frustrating, or actually very frustrating. ID 5, primigravida.*

The women expressed that it was exciting to wait and trust the body’s ability to start the process of giving birth. Thoughts and reflections about why the birth had not started and even whether there was something wrong with them emerged during the days or weeks of waiting.

*But that’s part of the charm with it all, although the difficulty is not knowing. …you have to trust the body to start it. ID 10, primigravida.*

### Unmet expectations

Despite the level of focus on the due date, the women perceived that the midwives failed to see and acknowledge them, particularly when overdue. Although they were aware of the availability of tests and examinations for term pregnancy, they were not offered any extra tests or examinations for overdue pregnancy and only received the standard care carried out in accordance with antenatal care guidelines. Almost all of the interviewed women reported a lack of information, and care that they felt they needed in their current situation. They wished to be seen and asked for their thoughts about PTP as opposed to just being told that induction of labour was being planned. In addition, they wished to be offered extra visits to the antenatal clinic.

*But above all, that they should have listen and asked more, instead of just providing information; ask how it feels without judging the feelings. It would have been enough for them just to listen and asked more. ID 3, primigravida.*

The women experienced contradictory feelings of hope and disappointment when the midwife told them that there would probably be no more visits. The feeling of hope was due to the fact that the birth was close, while disappointment arose when they were not asked about their feelings nor given information about what would happen in the event of a possible induction of labour.

*It’s like…maybe we’ll meet next week, but probably not, since you will probably have given birth by then; so, you’re hopeful, but no one talks about the actual feelings or gives information. ID 7, multigravida.*

The women also reported receiving no information concerning whether inducing labour was good or bad and instead were left to decide on the issue themselves.

*Because I sometimes get the impression that they don’t really say “This is what you should do”, which would have been good…that this is recommended, but instead they mostly leave it up to you. ID 1, primigravida.*

The women wondered about optimal timing for the midwife to provide information on possible induction of labour and considered that GW 35 or later was appropriate. In their view, if the information was provided early in the pregnancy it would not be understood in the same way or maybe forgotten altogether.

*Not too early, because I believe that one is not receptive at that time. But perhaps around GW 35 or so…. Then you begin to think more about the birth, before that you’re not really there. ID 7, multigravida.*

Part of not being seen and acknowledged by the midwife was described as the midwife’s failure to detect the women’s worry about something happening to the unborn child between weekly visits. Some of the women desired extra visits, not just for the welfare of the unborn child, but for themselves as well. Although they were worried and wondered why labour had not yet begun, they experienced a lack of interest and understanding on the part of midwives.

*I feel that the blood pressure, belly size and foetal heart rate examinations and tests as well as questions such as how I feel and if I take my iron tablets are not enough at this stage. Now that I’m overdue I feel I would like to know more than I did earlier in the pregnancy, such as how the unborn child actually feels, as it is so tight in there and I can feel it moving around in a lively manner……that is, the usual examinations don’t make me calm, although having said that I’m not really worried. ID 10, primigravida.*

### Seeking alternative sources of information

The women were unhappy with the failure of the midwife to provide information concerning the consequences of being overdue; what caused it and whether it implied risks for the unborn child or themselves. This resulted in them seeking other sources of information and they stated that nowadays it was natural to search for information on the Internet; to “Google” for answers to their questions. However, their experiences of seeking and finding answers were contradictory. While they were aware that not all information on the Internet was trustworthy or beneficial, they nevertheless found it interesting to read about how other women had experienced the same situation.

*But then I think it’s interesting to read about other women’s experiences. Even if it’s not scientific it’s still someone who has experienced it. And you can hear a little about how other expectant mothers experienced being overdue… it’s really something you want to hear about more than the medical aspects, that things are as they should be…or that it’s nothing abnormal. ID 9, primigravida.*

The women were aware of the importance of evaluating the sources of information on the Internet and being alert to ensure that the information did not influence their own experiences.

*…if I had had more basic information from the beginning I wouldn’t have Googled it. Then perhaps I might have got the right information immediately. ID 6, primigravida.*

The women reported that what they found on the Internet comprised difficult events and mainly negative experiences. Since the women themselves were uneasy about their condition, reading about such events only served to increase their fears.

*So, you just hear all these terrible stories. And then you feel worse. So, you have to be critical of the sources or otherwise you are lost. ID 8, primigravida.*

Another aspect of seeking information on the Internet was that the women did not always find what they sought, or receive answers to their questions.

*There is very, very little about the risks of being overdue, and very little about what you want to know. ID 2, multigravida.*

Other sources of information were people with personal experiences of pregnancy and birth including relatives, friends and acquaintances. The women mentioned that this strategy could be calming or lead to negative thoughts and that some conversations could trigger the notion that being overdue could be genetic.

*Both started spontaneously, so they told me to stay calm, it will happen, but you never know and begin thinking of the coming choice. ID 6, primigravida.*

*What was it like when I was born, is it hereditary or something? I have no idea if it is, but maybe a bit. If I came late maybe my child will be late too, or something. ID 10, primigravida.*

Having a partner or close relative such as a mother with whom to discuss these questions and thoughts was often important. Being able to discuss one’s thoughts with a partner could also have a calming effect.

*I talk a lot with my husband and even my mother and sister…and they’re pretty calm, so they calm me down pretty much and even the midwife has calmed me down. ID 6, primigravida.*

The women searched the Internet for tips that could start the labour. One woman who read that activity could start the labour said that she had been more active.

*This week I’ve become even more active because I want to get it started. ID 9, primigravida.*

### The influence of others

The women reported that it was not always easy coping with others’ questions, advice and curiosity about whether they had given birth. Being continuously connected to online social media, such as Facebook, and thereby being constantly asked if they had given birth, could be experienced as stressful. They felt that the people around them wanted to be informed as soon as possible, as they were also impatient for the child to arrive. If they had not updated Facebook for a few hours, people would phone. Answering the telephone and even making phone calls oneself could be stressful when the first question was about whether the child was born or if the delivery had begun.

*I can’t phone twice…I say to myself, oh, now I’ve phoned my mum twice within a short space of time, she’ll think that something has happened, so I have to say right away that nothing has happened. ID 9, primigravida.*

Even phoning relatives and friends was stressful; as they expected that the child had arrived and thought that the phone call was to inform them of this. A week after the due date the women experienced that relatives became calmer. They did not phone as often to ask if the child had arrived. A reason for this could be that the women had repeatedly said that they would let them know when the child was born. However, some continued to phone, which led to irritation and increased frustration for the women about not having given birth.

*…and the week before and a few days after, every time I phoned they thought that something had happened, but now they’ve stopped. ID 9, primigravida.*

The women also felt guilty about people in their surroundings because they knew that the individuals in question cared about them. This guilty conscience was due to the women not having the strength to talk to anyone and say that the child had not come or discuss why it had not arrived.

The women looked forward to being able to truly surprise friends and relatives with the news of the child’s birth, but felt it would be impossible due to constantly having to update their pregnancy status via social media. If the women did not answer the telephone or log on to social media**,** they believed that others would immediately begin to speculate that the delivery had begun or that the child had arrived.

*So as far as coming as a surprise that the child has arrived, I can forget it… as soon as I don’t answer the phone the speculation begins. ID 6, primigravida.*

An important aspect was that the partner felt a part of the pregnancy and the present situation so that they could support and calm each other by talking through their thoughts together.

*so that.. it’s the two of us...that we stay calm and can help each other in this too and that this is… really nice… since it’s so important to feel that we’re doing this together. ID 1, primigravida.*

A negative feeling was jealousy that their partners were able to go to work every morning; a job to go to where the partner could think of something other than the pregnancy. The women experienced being left home alone without the strength to do what they had planned.

*That you go around all day and have nothing to do, while he can go to work and think about something else. Yes, go out and do things because he can move about and I can’t. And here I am. ID 5, primigravida.*

The women experienced that their partners also had a difficult time during the wait. They believed their partner could experience worry, tension and impatience for the labour to begin. They also felt that their partner’s motivation to leave in the morning was reduced or had disappeared.

*It feels a bit like all conversations return to this discussion, the lack of progress in some way and he finds it really difficult to go to work as his job has no real meaning now and he just goes to his workplace and waits. ID 10, primigravida.*

## Discussion

The main findings in this study were that the pregnant women who had passed GW 41 + 0 felt that they were left in limbo, a void characterised by contradictions related to time, giving birth and the condition.

Being in limbo meant that the women experienced their existence as dominated by negative feelings that increased over time. Eri et al. [43] revealed that women moved into a “waiting” mode and a state of constant physical alertness around the due date. However, in our study this “waiting” changed in a negative way over time from trust in to distrust of the body’s ability to give birth. Earlier research has highlighted the importance of supportive professional encounters during pregnancy which women can develop trust in the capacity of their own body and feelings of control [32, 44]. Therefore, the midwife has a central role in supporting these women in trusting their bodies during both pregnancy and birth. During the prenatal and the natal period, greater continuity of care, woman oriented communication and a personal approach with enough time for each woman could enhance the care [45–49].

According to the findings of this study, the women wished labour to start spontaneously and during the pregnancy they trusted their body to achieve just that. However, after GW ≥41 they started to doubt their body’s capacity, which was mentally exhausting. At the same time, they were aware that very few women actually give birth on the estimated date of delivery. However, even though they knew this intellectually, it was difficult for them to ignore the negative feelings that arose. This is supported by previous research [17, 45] that also revealed that women in a late term pregnancy can suffer from stress and worry. The women experienced how healthcare during pregnancy was planned until the estimated date of delivery and were therefore surprised when visits, tests and examinations proceeded as usual after the estimated date of delivery. Ayers et al. [17] demonstrated that focus on the estimated date of delivery may decrease if antenatal care professionals give the women the latest instead of the estimated delivery date. This means that if the woman has not giving birth by the latest date, she will be induced. This might be a good strategy for diverting the focus away from the estimated delivery date. This strategy should be complemented by a greater interest in the woman’s experience. Previous research has revealed that seeing the woman and allowing her to participate is important for enabling a feeling of control during pregnancy as well as reinforcing her trust in the ability of her own body to manage pregnancy and birth [44, 50]. This will hopefully prevent the feeling of being in limbo by promoting more positive emotions and greater trust in the body’s ability to give birth.

The state of limbo made “just being” difficult for the women, as they experienced a loss of control over their body, the pregnancy and the birth. However, losing control of the situation was also positive as they learned to accept that they could not control how or when labour would begin. Feelings of control have been described as important for a positive birth experience by Bondas and Eriksson [45]. The challenge for healthcare professionals is to understand the generation of women who are now giving birth and whether there is a difference compared to 10-15 years ago. Today social media play an important role in influencing the women. Lundgren has previously demonstrated the importance of individual support during pregnancy and birth for helping the women to feel secure [44]. It is possible that women who search for and find information via social media require more individual support rather than more information.

Our study shows that the women sought support from their midwives but experienced a lack of acknowledgement and support, as the midwives tended to define overdue pregnancy as normal, thus failing to confirm the women’s experiences. However, women reported lacking such support during induction of labour and described care without caring [39], which is also consistent with the experiences of pregnant women with a systemic disorder and women with pregnancy-related pelvic girdle pain [51–53].

Understanding the Swedish maternity care system is of importance when interpreting the findings and it is necessary to point out that in Sweden there are different care providers for antenatal care and in the hospital. This can explain the experience of a gap in the care chain, as no extra check-up of either the woman or the unborn child takes place during this period. Continuity of care has been shown to influence patient satisfaction [54]. This lack of continuity of care can strengthen the sense of being in limbo. One question for further discussion is whether pregnant women should be offered a meeting with a professional from the hospital/delivery ward to receive information about the induction of labour in order to ensure a feeling of security and preparedness to give birth.

Although the women stated that their bodies were ready for labour, they did not know whether the baby was ready to be born. They wished that labour would start naturally but knew that it would be induced 2 weeks after the estimated date of delivery. They thought about a possible induction and felt it had been decided that labour would be induced if it did not begin spontaneously. Previous research has shown that women with post term pregnancies believed that neither their body nor the foetus was ready for birth [38]. The situation of these women could be improved by greater continuity on the part of the healthcare professionals during the prenatal period, woman centred communication and a personal approach including sufficient time devoted to each woman [45–48].

This study reveals that women who had reached GW 41 lacked information about care and management during the final days of the pregnancy. Dissatisfaction with antenatal care, lack of information and pregnant women’s experiences of not being taken seriously have been previously described [47]. In addition, the following prenatal care outcomes have been found to be important: reducing pregnancy-related stress and worries; developing confidence and knowledge to improve health; preparing for labour and birth; having supportive relationships and participating in decision making about the induction process [38, 49]. These factors provided greater satisfaction and a better birth experience [55]. Lack of information led to the women searching for information on the Internet [56–58]. However, our study indicates a risk that discussions with relatives, workmates and friends on the Internet only serve to intensify the pregnant woman’s feelings of uncertainty and fear, rather than providing relief. This was also found in a published article from Denmark were the informants experienced that people around them had a negative influence in PTP [40]**.** Nevertheless, the women in our study expressed that the information they found on the Internet was positive in that they understood that they were not alone in experiencing negative feelings due to passing the estimated date of delivery. It is essential that healthcare professionals guide pregnant women to high quality web-based information and take time to discuss it at antenatal visits [58].

### Limitations of the study

The phenomenological approach was deemed most suitable for gaining a deeper understanding of PTP. The strength of this approach is that it provides a possibility to delve more deeply into the nuances and variations of a specific phenomenon. The aim of this study was to obtain a deeper understanding of women’s experiences of a PTP. As with all qualitative research, the findings cannot be considered universal, but rather contextual and relevant to a specific phenomenon and context [41], in this case Swedish women in a Swedish maternity care setting. The criterion for achieving trustworthiness is to carefully show the steps in the analysis so that the reader can follow the work with the text and its meaning, which we tried to do during the research process. The range of ages and socioeconomic levels of the women who were interviewed increased variations. The study did not include women from low income groups or from cultures other than the Swedish one, which can be a limitation. However, the fact that the findings are contextual does not mean that they lack relevance in other contexts. When transferring the findings*,* the new context must be considered.

The first author (AW) is a midwife with long experience of supporting pregnant women during and after childbirth. Thus, her preunderstanding had to be bridled throughout the interviews and data analysis process [41]. In addition, she was not known to the participating women before the interviews.

## Conclusion

Women’s experiences of pregnancy at ≥41 GW can be understood as being in a state of limbo, a time of unexpected waiting. The time of waiting consisted of contradictions related to time, giving birth and their condition, with increasingly negative feelings and mistrust of the body’s ability to give birth. The contradictions became more evident as the pregnancy progressed, especially their mistrust to the body’s ability to start the labour. When in limbo the women were kept waiting without sufficient information and support from their midwives, which meant that they had to deal with uncertain information from the Internet on their own. Without information about PTP the women sometimes had to trust the advice coming from relatives and other people close to them.

It is vital that these women are seen and acknowledged by midwives at antenatal clinics, in addition to being asked how they experience waiting for delivery. It is important that information on the management of the pregnancy and upcoming labour provided at the antenatal unit and the maternity ward is consistent. Offering an extra visit to the antenatal clinic can be a means of reducing worry and the feeling of being in a state of limbo. Midwifery support should be based on trust, confidence, familiarity, mutual respect, shared decision-making and good communication. The study did not examine what would have changed women’s experiences and whilst it is useful to discuss the implications of the findings for practice, more research is needed to know which aspects of care could be improved*.*

## Abbreviations

ACOG: American College of Obstetricians and Gynaecologists
DSOG: The Danish Society of Obstetrics and Gynaecology
GW: Gestational week
PTP: Post term pregnancy
RCOG: Royal College of Obstetricians and Gynaecologists in Great Britain
RCT: Randomised controlled trial
SCOG: The Society of Obstetricians and Gynaecologists of Canada
SU: Sahlgrenska University Hospital
WHO: World Health Organisation
